# Integrating Multiparametric MRI and PSMA PET Imaging in Prostate Cancer: Toward a Unified Diagnostic and Risk-Stratification Paradigm

**DOI:** 10.3390/medicina62030610

**Published:** 2026-03-23

**Authors:** Rosa Alba Pugliesi, Roberto Cannella, Karim Ben Mansour, Daniele Di Biagio, Pierpaolo Alongi

**Affiliations:** 1 Department of Biomedicine, Neuroscience and Advanced Diagnostics (BiND), University of Palermo, Via del Vespro 129, 90127 Palermo, Italypierpaolo.alongi@unipa.it (P.A.); 2 Department of Radiology, Hôpital de Morges, Chemin du Crêt 2, 1110 Morges, Switzerland; karim.ben.mansour@hotmail.com; 3 Nuclear Medicine Centre, San Pietro Fatebenefratelli Hospital—Express Diagnostics, Via Cassia 600, 00189 Rome, Italy; d.dibiagio@expressdiagnostic.it

**Keywords:** prostate cancer, multiparametric magnetic resonance imaging (mpMRI), prostate-specific membrane antigen PET (PSMA PET), hybrid imaging, precision medicine

## Abstract

Prostate cancer represents a highly prevalent malignancy affecting men globally, necessitating precise staging and risk stratification for effective patient management. Multiparametric magnetic resonance imaging (mpMRI) and prostate-specific membrane antigen positron emission tomography (PSMA PET) have individually revolutionized the diagnosis and management of prostate cancer. Recent developments emphasize the integration of these imaging modalities to improve detection capabilities, inform therapeutic interventions, and facilitate personalized management. This narrative article reviews existing literature on the clinical utilization of mpMRI and PSMA PET in prostate cancer. Key areas encompass initial diagnosis, both local and systemic staging, detection of biochemical recurrence, and their influence in treatment strategies. The integration of mpMRI and PSMA PET offers complementary insights, with mpMRI demonstrating superior capability in local tumor characterization and PSMA PET enhancing the detection of nodal and distant metastases. Quantitative imaging biomarkers, including apparent diffusion coefficient (ADC) and standardized uptake values (SUV), have the potential to improve risk stratification and inform personalized treatment strategies. Hybrid imaging techniques may improve diagnostic accuracy and guide decisions regarding surgery, radiotherapy, and systemic treatment. The integration of mpMRI and PSMA PET allows a potentially transformative advancement in the realm of precision imaging for prostate cancer. This integrated approach can improve diagnostic accuracy, better define disease extent, and support personalized management strategies.

## 1. Introduction

Prostate cancer is one of the most frequently diagnosed cancers among males worldwide. In 2022 there were an estimated 1,466,680 new cases of prostate cancer globally and about 396,792 deaths attributable to the disease, making it the second most commonly diagnosed cancer in men and the fifth leading cause of cancer death worldwide [1,2,3]. Based on recent international cancer statistics, prostate cancer is responsible for over 1.4 million new cases each year, with significant regional differences in incidence and prognosis [4,5]. Although many patients are diagnosed with indolent disease, a substantial proportion present with or develop clinically aggressive cancer, highlighting the heterogeneity of prostate cancer biology [6]. This variability requires precise risk stratification to effectively manage the competing risks of overtreatment and undertreatment. Current management strategies are progressively dependent on imaging-based evaluations to guide diagnosis, staging, and personalized treatment [7].

Standard diagnostic approaches for prostate cancer typically involve prostate-specific antigen (PSA) testing, digital rectal examination, systematic transrectal ultrasound-guided biopsy, and imaging techniques such as computed tomography and bone scintigraphy [8]. However, these exams have inherent limitations such as biopsy sampling error that may underestimate tumor grade or overlook clinically significant lesions [9]. Furthermore, conventional imaging demonstrates limited sensitivity in detecting early osseous metastases, thereby restricting its effectiveness for precise staging [10]. These limitations have prompted the implementation of advanced imaging modalities that offer improved anatomic localization and detailed biological characterization of disease [11]. 

Progress in prostate imaging over the last decade has transformed the diagnostic landscape, with multiparametric MRI (mpMRI) and prostate-specific membrane antigen positron emission tomography (PSMA PET) being the main imaging modalities for the diagnosis and staging [12]. mpMRI has demonstrated high diagnostic performance in intraprostatic tumor detection and local staging relative to conventional imaging techniques, whereas PSMA PET has demonstrated significantly greater sensitivity for identifying nodal and distant metastatic disease [13]. Increasing evidence suggests that these techniques provide complementary information across clinical scenarios.

Importantly, current standard staging pathways generally apply mpMRI for local assessment and reserve PSMA PET primarily for high-risk staging or biochemical recurrence [14]. In contrast, the integrated paradigm discussed in this review proposes coordinated interpretation of both modalities across defined clinical scenarios to refine risk classification and therapeutic planning. This conceptual distinction between sequential use and structured integration underpins the analytical framework of the present review.

This narrative article reviews existing literature on mpMRI and PSMA PET in prostate cancer and critically evaluates their complementary integration within contemporary risk stratification models.

Although designed as a narrative review, a structured literature search was performed to enhance transparency. PubMed/MEDLINE, Embase, and Web of Science were searched for studies published between January 2015 and January 2026 using predefined keywords (Supplementary Table S1). Priority was given to randomized trials, prospective multicenter studies, meta-analyses, and guideline-defining publications. Exploratory single-center studies were included when addressing emerging topics such as radiomics or artificial intelligence. Non-English articles, case reports, and studies lacking histopathologic or clinical validation were excluded.

## 2. Multiparametric MRI (mpMRI) in Prostate Cancer

### 2.1. Technical Considerations

Multiparametric MRI combines high-resolution T2-weighted imaging with functional techniques, such as diffusion-weighted imaging (DWI) and dynamic contrast-enhanced (DCE) imaging, to characterize prostate lesions at both the anatomical and cellular levels [15]. Among these sequences, DWI and the apparent diffusion coefficient (ADC) are the most relevant tools, as decreased ADC values are associated with increased tumor cellularity and a higher histopathologic grade [16]. Standardized interpretation is achieved through structured reporting systems such as the PI-RADS that include lesion morphology, diffusion properties, and presence of enhancement, allowing for consistent lesion evaluation and risk stratification. These technological advancements have established multiparametric MRI (mpMRI) as an integral component of contemporary prostate cancer imaging.

### 2.2. Clinical Applications of mpMRI

mpMRI is essential in the detection, localization, and characterization of primary prostate cancer [17]. Prospective studies and meta-analyses have shown that mpMRI-guided biopsy improves the detection of clinically significant prostate cancer while decreasing unnecessary biopsies and the overdiagnosis of low-risk conditions [18]. Beyond diagnosis, mpMRI remains the imaging modality of choice for local staging, offering essential insights into extracapsular extension, seminal vesicle invasion, and the tumor’s proximity to neurovascular structures [19]. These characteristics directly impact surgical planning, the delineation of radiotherapy targets, and eligibility criteria for focal therapies or active surveillance strategies [18].

Despite the high diagnostic performance of mpMRI, histopathologic confirmation through biopsy remains the reference standard for prostate cancer diagnosis and grading [20]. Imaging findings, whether derived from mpMRI, PSMA PET, or integrated approaches, must therefore be interpreted in conjunction with targeted or systematic biopsy results to establish definitive ground truth.

### 2.3. Limitations of mpMRI

Although it is well-established, false-negative results may arise in cases involving small-volume tumors, low-grade malignancies, or lesions exhibiting limited diffusion restriction [21]. Additionally, diagnostic accuracy may be diminished within the transition zone [22]. Interobserver variability continues to be a concern, especially outside high-volume centers, and image quality is affected by scanner technology and acquisition parameters [21]. Recent comparative studies indicate that certain biologically significant tumors may display minimal conspicuity on mpMRI while showing pronounced uptake on PSMA PET, emphasizing the importance of employing complementary imaging modalities rather than depending solely on a single technique [11].

## 3. PSMA PET Imaging in Prostate Cancer

### 3.1. Technical Overview

Prostate-specific membrane antigen (PSMA) PET imaging capitalizes on the significant overexpression of PSMA on prostate cancer cells, particularly in high-grade and metastatic disease. In clinical practice, PSMA PET is most frequently conducted using hybrid PET/CT or PET/MRI systems with small-molecule PSMA-targeted radioligands labeled with either 68Ga (e.g., 68Ga-PSMA-11) or 18F (e.g., 18F-DCFPyL/piflufolastat, 18F-rhPSMA-7.3/flotufolastat, 18F-PSMA-1007). Local availability, operational considerations, and tracer-specific biodistribution characteristics are the primary factors that influence the selection of a tracer [23,24,25,26].

Overall, PSMA PET tracers demonstrate broadly comparable diagnostic performance; therefore, tracer selection is primarily driven by logistical factors and known biodistribution-related pitfalls rather than consistent differences in diagnostic accuracy [22,27,28]. While 68Ga-based tracers are widely accessible, 18F-labeled tracers offer superior spatial resolution [27]. In addition, 18F-PSMA-1007 is characterized by reduced urinary excretion, which may improve evaluation of the prostate and prostate bed [29]. Among 68Ga tracers, 68Ga-PSMA-11 remains the most extensively validated tracer and benefits from on-site generator-based production; however, its short physical half-life and limited generator yield may constrain patient throughput and scheduling flexibility [23,30]. Following intravenous administration, whole-body imaging with 68Ga-PSMA-11 is typically initiated approximately 50–70 min post-injection, with delayed pelvic acquisitions performed selectively to improve lesion conspicuity [31,32]. In comparison, 18F-labeled PSMA tracers provide greater distribution efficiency and scheduling flexibility due to centralized cyclotron production and a longer half-life, as well as improved physical imaging properties. When identical targeting vectors are employed, 18F-PSMA-11 has demonstrated non-inferiority to 68Ga-PSMA-11 in a prospective randomized crossover trial [24,33]. Among available 18F agents, 18F-DCFPyL (piflufolastat) is supported by robust phase III evidence, particularly in the setting of biochemical recurrence. Conversely, 18F-PSMA-1007 may facilitate improved assessment of the pelvis and prostate bed owing to low urinary activity but is associated with interpretation challenges, including nonspecific uptake in benign bone lesions [34,35].

Hybrid PET/MRI may be preferred when superior soft-tissue contrast is required for precise local tumor localization and evaluation of extracapsular extension or seminal vesicle invasion, whereas PET/CT remains the most widely utilized modality for whole-body staging [36,37,38,39].

Image reconstruction is typically performed using iterative algorithms, such as ordered-subset expectation maximization (OSEM), with increasing adoption of regularized reconstruction techniques (e.g., block-sequential regularized expectation maximization, BSREM), particularly on digital time-of-flight (TOF) systems, to reduce image noise while preserving lesion detectability and quantitative accuracy [32]. Quantitative metrics, including standardized uptake values (SUVs) and tumor-to-background ratios, provide semi-quantitative measures of tracer uptake and have been associated with tumor burden and biological aggressiveness [39]. However, the establishment of standardized quantitative thresholds remains an area of ongoing investigation [40].

### 3.2. Clinical Applications of PSMA PET

PSMA PET has shown improved diagnostic accuracy relative to conventional imaging modalities in identifying nodal and distant metastases [25]. During the initial staging procedure, PSMA PET has demonstrated clinically meaningful diagnostic accuracy for primary staging and biochemical recurrence, including superiority over conventional imaging for detecting nodal, even when they are smaller than one centimetre, and distant metastases in high-risk disease [13,40,41,42]. Its significance is especially recognized in cases of biochemical recurrence, where PSMA PET effectively detects disease at low PSA levels, often leading to alterations in clinical management [43]. Prospective evidence shows high positive predictive value and PSA-dependent detection, supporting its use to localize disease when salvage strategies are being considered [39,44,45]. Head-to-head comparisons indicate that PSMA PET generally outperforms conventional imaging and can outperform other PET tracers (e.g., fluciclovine, choline) in clinically relevant recurrence settings, informing tracer choice when PET is pursued for management decisions [46,47,48]. Current studies have demonstrated that PSMA PET findings commonly result in modifications to treatment strategies, such as incorporating metastasis-directed therapy, adjusting radiotherapy fields, or initiating systemic therapy [49,50]. Consequently, PSMA PET has become a fundamental component in contemporary protocols for the staging and restaging of prostate cancer. For reporting, standardized interpretation frameworks (e.g., PROMISE miTNM, PSMA-RADS, and EANM-aligned procedure/reporting guidance) are increasingly used to improve reproducibility and multidisciplinary communication [51].

### 3.3. Limitations of PSMA PET

Despite its high sensitivity, physiological uptake in organs such as the salivary glands, kidneys, liver, and sympathetic ganglia may result in false-positive findings and confound image interpretation [52]. Furthermore, certain prostate malignancies, particularly those that are low-grade or neuroendocrine-differentiated, may exhibit minimal or no PSMA expression, which can lead to false-negative imaging results [53]. The detection of diseases that involve very small volumes continues to present significant challenges, and partial-volume effects may impede precise quantification [53].

The major unresolved limitations of clinical PSMA PET are as follows: (1) false positives, particularly isolated or equivocal bone uptake due to non-prostatic PSMA expression/benign causes, and (2) limited sensitivity for micrometastatic disease below PET resolution, particularly in pelvic lymph nodes, so a negative scan cannot exclude microscopic spread [27,40,54].

## 4. Integration of mpMRI and PSMA PET: Toward a Unified Diagnostic and Risk-Stratification Paradigm

In clinical practice, the integrated paradigm is best conceptualized across specific clinical scenarios: (1) primary diagnosis in biopsy-naïve patients, (2) initial staging in intermediate–high-risk disease, (3) biochemical recurrence, and (4) treatment planning for surgery or radiotherapy. Framing integration within these distinct scenarios allows clearer differentiation of evidence strength and clinical utility.

The integrated use of mpMRI and PSMA PET/CT takes advantage of the complementary strengths of high-resolution local anatomic imaging and whole-body molecular staging to improve the detection and characterization of intraprostatic disease, nodal spread, and distant metastases in intermediate–high-risk prostate cancer patients [27,52,55,56]. Meta-analyses and comparative studies have demonstrated that PSMA PET/CT generally shows higher pooled sensitivity than mpMRI for lymph node metastasis detection (74% vs. 45%) with similarly high specificity (96% vs. 92%) when pelvic lymph node dissection is used as the reference standard [57]. Moreover, other pooled analyses in intermediate–high-risk cohorts report that 68Ga-PSMA PET/CT achieves sensitivities in the range of approximately 65–74% and specificities of 94–97% for nodal staging, compared with lower or variable sensitivities for mpMRI [58]. This evidence suggests that combining mpMRI and PSMA PET/CT can enhance overall diagnostic performance and provide greater confidence in staging decisions than either modality alone.

By distinguishing between localized and systemic prostate cancer, this boosts diagnostic precision, refines risk stratification, and more effectively informs treatment decisions [59]. This comprehensive approach is consistent with the emergence of radiomics-based models that utilize multimodal imaging to characterize tumor phenotype and aggressiveness in a manner that surpasses traditional staging systems [60] (Table 1).

mpMRI and PSMA PET offer unique and complementary information [61]. mpMRI is capable of accurately detect prostate lesions, characterize lesions, and evaluate extracapsular extension or seminal vesicle invasion, thereby assisting in the planning of local treatment [60]. In the case of the primary tumor, mpMRI remains the reference standard for local staging (e.g., assessment of extracapsular extension or ECE and seminal vesicle invasion or SVI) [62]. However, PSMA PET can provide additional value in tumor localization and risk stratification, particularly when mpMRI is ambiguous [63]. This supports combined interpretation and, when feasible, PET/MRI strategies [27,36,46,64]. PSMA PET enables thorough whole-body staging through tumor-specific molecular targeting, allowing for the detection of nodal and distant metastases [24]. For pelvic nodal staging, PSMA PET/CT offers higher accuracy than conventional imaging but still misses micrometastases, so its main advantage is identifying clinically actionable N disease beyond CT/MRI size criteria rather than replacing surgical nodal staging when indicated [39,46,52,65].

For distant metastases, PSMA PET/CT improves detection versus conventional imaging and can upstage patients with otherwise “localized” disease, enabling more appropriate systemic and/or metastasis-directed management selection [46,66]. The complementary strengths of mpMRI and PSMA-PET are summarized in Table 2.

**Figure 1:**
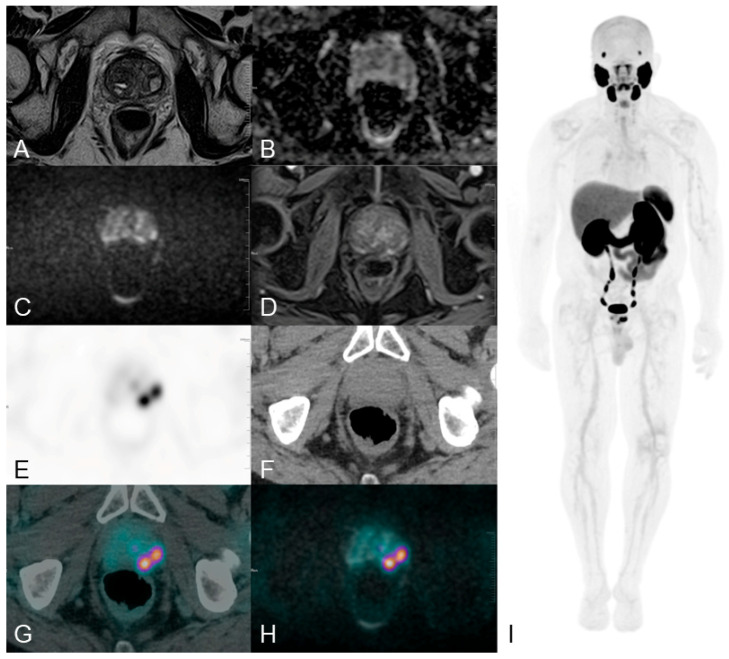
MRI and PSMA-PET/CT in prostate cancer staging. Long Axial Field-of-View (LAFOV) Total-Body PET/CT (194 cm uEXPLORER, United Imaging, Shanghai, China) with 18F-Piflufolastat (2.64 MBq/kg) in a patient with Gleason 8 (4 + 4) and PSA 14.2 ng/mL. Panels: (**A**) T2-weighted MRI; (**B**) ADC; (**C**) high b-value DWI; (**D**) contrast-enhanced T1; (**E**) attenuation-corrected PSMA-PET; (**F**) low-dose CT; (**G**) fused PET/CT; (**H**) PET/MRI-DWI fusion; (**I**) PET MIP. PET tracer distribution is visualized using the ‘Warm-Metal’ linear color scale. Image reconstruction and post-processing were performed using United Imaging Healthcare PET/CT and CT Image Post-processing Software (uWS-MI:R002.32.0.1749877; uWS-CT:F006.34.0.1749877). Higher metabolic activity or tracer avidity is represented by the transition from cooler tones (black/green) to warmer tones (orange/white). MRI shows a left-lobe lesion with mild diffusion restriction; PET identifies two tracer-avid areas, including a posterior focus near the capsule and rectal wall not clearly seen on MRI. MRI and PET complement each other for accurate local staging. Images courtesy of San Pietro Fatebenefratelli Hospital, Rome.

Inconsistent results are often encountered due to differences in sensitivity to tumor volume, grade, and biological features. Integrated imaging improves local staging and surgical risk assessment by reducing ambiguous interpretations and elevating staging accuracy [62]. This is achieved by using multiparametric MRI to identify the primary intraprostatic lesion and PSMA PET to detect additional capsular-adjacent PET-avid foci (Figure 1).

It is indispensable to recognize that the discrepancy between the findings of mpMRI and PSMA PET should not be read as a mere limitation of either modality, but rather as an indication of the heterogeneity of the underlying tumor [65]. In contrast, certain MRI-conspicuous tumors may exhibit low PSMA uptake as a result of variable target expression or dedifferentiation, while lesions with low cellular density or limited diffusion restriction may express high PSMA [67]. It is imperative to acknowledge these biological distinctions in order to prevent misinterpretation and to substantiate the case for the use of combined imaging over sequential or competitive methods [68].

**Figure 2:**
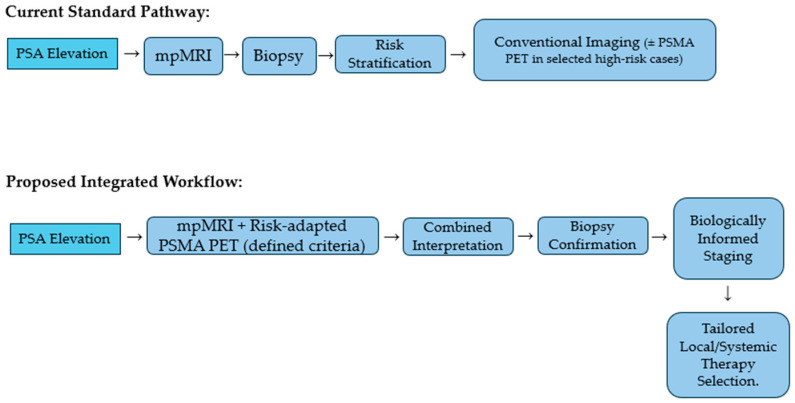
Decision-Flow Framework for Integrated Imaging. This schematic comparison clarifies that the proposed paradigm does not replace established diagnostic standards but seeks to harmonize anatomical and molecular data earlier in the decision process.

As illustrated in Figure 2, the schematic comparison clarifies that the proposed paradigm does not replace established diagnostic standards but seeks to harmonize anatomical and molecular data earlier in the decision process. The integration framework emphasizes structured interpretation rather than parallel, uncoordinated test utilization.

Integrated mpMRI and PSMA PET enable the reclassification of clinically significant risks across diagnostic, recurrent, and treatment-planning settings (Table 3).

**Figure 3:**
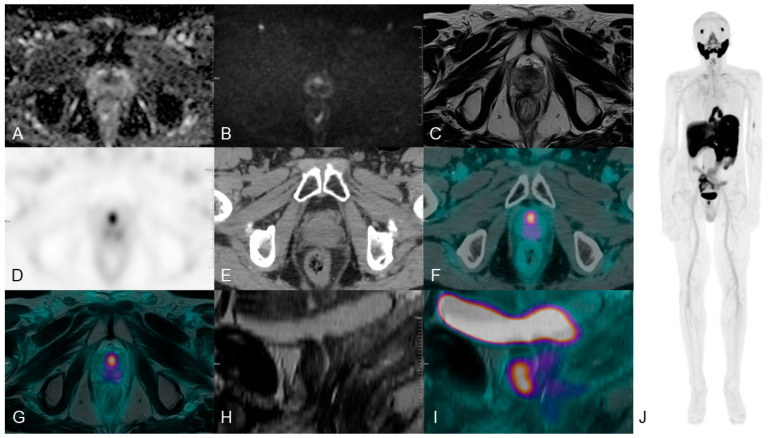
MRI and PSMA-PET/CT in intra-prostatic relapse evaluation. Long Axial Field-of-View (LAFOV) Total-Body PET/CT (194 cm uEXPLORER, United Imaging) with 18F-Piflufolastat (3.35 MBq/kg) in a patient with PSA 3.98 ng/mL, Gleason 6 (3 + 3), post-radiotherapy. Panels: (**A**) ADC; (**B**) high b-value DWI; (**C**) T2-weighted MRI; (**D**) attenuation-corrected PSMA-PET; (**E**) low-dose CT; (**F**) fused PET/CT; (**G**) axial PET/T2 fusion; (**H**) sagittal T2; (**I**) sagittal PET/T2 fusion; (**J**) PET MIP. PET tracer distribution is visualized using the ‘Warm-Metal’ linear color scale. Image reconstruction and post-processing were performed usingUnited Imaging Healthcare PET/CT and CT Image Post-processing Software (uWS-MI:R002.32.0.1749877; uWS-CT:F006.34.0.1749877), where higher metabolic activity or tracer avidity is represented by the transition from cooler tones (black/green) to warmer tones (orange/white). MRI shows a focal lesion with diffusion restriction and T2 hypointensity, matching PET uptake along the anterior midline. MRI clarifies urethral position, confirming true intraprostatic relapse. Complementary MRI-PET improves lesion characterization. Images courtesy of Nuclear Medicine Centre, San Pietro Fatebenefratelli Hospital, Rome, Italy.

In patients without prior biopsy, integrated imaging improves the identification of clinically significant intraprostatic pathology while efficiently ruling out occult metastases, thereby reducing the risk of understaging [71]. In recurrence, multiparametric MRI allow to confirm PSMA PET findings by demonstrating the presence of tumor recurrence with diffusion restriction (Figure 3), thereby providing direct guidance for treatment planning.

In patients with intermediate- and high-risk disease, PSMA PET frequently identifies occult nodal involvement, leading to upstaging and adjustments in surgical or radiotherapy approaches [65]. During biochemical recurrence, integrated imaging improves the capacity to differentiate between local and metastatic disease, thereby informing decisions related to salvage or systemic treatment strategies [61].

Importantly, while integrated imaging improves staging confidence, it does not yet constitute a validated, outcome-driven unified risk-stratification system. Prospective randomized evidence demonstrating survival benefit or long-term outcome modification based specifically on combined mpMRI and PSMA PET remains limited [69]. Trials such as proPSMA [13] have established the superiority of PSMA PET over conventional imaging for staging accuracy, yet equivalent high-level trials directly testing combined mpMRI–PSMA paradigms are still lacking. Therefore, integration should currently be regarded as a complementary strategy rather than a fully established replacement for existing staging frameworks.

Cost-effectiveness analyses [72,73] suggest potential downstream savings through improved staging accuracy; however, real-world accessibility, radiotracer availability, infrastructure requirements, and reimbursement policies remain substantial barriers in many healthcare systems. These considerations temper the immediate generalizability of integrated imaging approaches.

## 5. Impact on Clinical Management

Accurate preoperative localization and staging are essential for achieving optimal surgical outcomes in prostate cancer [74,75]. New evidence suggests that the inclusion of PSMA PET, particularly in intermediate- and high-risk patients, can elevate surgical planning by detecting occult nodal disease that is not detected on MRI or conventional imaging [76,77]. In the post-prostatectomy setting, the combination of mpMRI and PSMA PET interpretation has also been demonstrated to improve the localization of local recurrence, which has the potential to contribute to salvage surgery or targeted interventions [78].

In order to optimize tumor control while minimizing adverse effects, radiotherapy necessitates precise target delineation and risk-adapted dose escalation. mpMRI provides high-resolution anatomical detail for defining dominant intraprostatic lesions, which has enabled focal boost strategies within the prostate gland [79]. PSMA PET complements this approach by identifying involved lymph nodes and distant metastases, frequently altering radiation fields and treatment intent [80]. Changes in radiotherapy planning, such as expanded nodal coverage or stereotactic treatment of oligometastatic disease, have been associated with integrated imaging approaches, thereby supporting a more personalized radiation strategy [70]. This has facilitated the prompt identification of individuals who could benefit from systemic medications, like androgen deprivation therapy or innovative androgen receptor-targeted agents [81]. Upon interpretation in conjunction with mpMRI, PSMA PET boost the ability to differentiate between localized and systemic disease, thereby facilitating the informed escalation or deescalation of systemic therapy and bolstering contemporary strategies, such as metastasis-directed treatment, within evolving therapeutic paradigms [82]. The link between tumor aggressiveness and clinical outcomes, as shown by quantitative biomarkers from mpMRI (e.g., ADC) and PSMA PET (e.g., SUV), suggests refined risk classification [83].

Combining imaging to characterize disease extent and biology may reduce overtreatment of indolent disorders and help intensify aggressive cancer treatment [84]. Integrated imaging may enable advanced, patient-specific care regimens, although more validation is needed before widespread clinical use [85].

## 6. Challenges and Future Directions

The absence of standardized acquisition and interpretation protocols is a substantial impediment to the widespread clinical implementation of integrated mpMRI and PSMA PET [86]. Although there is still a significant amount of variation between centers and readers, PI-RADS has improved the consistency of mpMRI reporting [87]. The necessity for standardized composite imaging methods and reporting platforms to ensure reproducibility and facilitate multicenter research is underscored by the fact that PSMA PET interpretation criteria and quantitative thresholds vary among radiotracers and institutions [88]. The EANM E-PSMA recommendations for acquisition and interpretation, PROMISE v2 with the 5-point PRIMARY score for local disease characterization and prognostic stratification, and PSMA-RADS for lesion-level likelihood classification and handling of equivocal findings are all part of the current frameworks for standardizing integrated reporting and multidisciplinary communication [50,89,90].

Despite promising prospective and retrospective findings, evidence remains limited, largely from small or single-center studies, underscoring the need for multicenter trials assessing accuracy and patient-centered outcomes [91].

Emerging total-body/Long Axial Field-of-View (LAFOV) Total-Body PET/CT systems, due to their significantly heightened sensitivity, may further augment PSMA PET/CT for primary tumor evaluation and improve the detection of very small-volume (micrometastatic) nodal disease, potentially alleviating (though not completely resolving) the acknowledged sensitivity deficiency of current PSMA PET for pelvic lymph node micrometastases [12,38,50].

Advanced analytical methodologies, encompassing radiomics, machine learning, and artificial intelligence, are progressively being investigated to amalgamate multiparametric imaging data [92]. Preliminary research indicates that the integration of quantitative mpMRI and PSMA PET characteristics may expand predictive modeling for tumor aggressiveness and therapeutic response [93]. Nevertheless, these methods are experimental and require rigorous validation, homogeneous feature extraction, and explicit model development before clinical use [94]. Theranostic potential is another mpMRI/PSMA PET imaging use. PSMA PET can identify patients with sufficient target expression and exclude those with PSMA-negative or heterogeneous illness utilizing PSMA-targeted radioligand treatment, such as 177Lu-PSMA [22]. mpMRI can detect tumor heterogeneity by revealing clinically significant but non-PSMA-avid intraprostatic components and local tumor extent. PSMA-based theranostic patient selection, combined local and systemic therapy selection, and individualized treatment sequencing can be optimized by integrated imaging [95].

Finally, cost, availability, and access still prevent broad use of advanced imaging. Due to infrastructural and radiotracer requirements, PSMA PET is limited in resource-constrained situations [96]. Cost-effectiveness assessments that account cascade effects on treatment choices and outcomes are needed to deploy integrated imaging solutions in healthcare systems [68,97].

Beyond imaging, integration with genomics, radiogenomics, and other omics-based biomarkers may facilitate more robust and generalizable risk stratification [98]. Preliminary radiogenomic studies suggest correlations between imaging phenotypes and molecular alterations, including PTEN loss and genomic risk classifiers [99]. Such integrative approaches may ultimately bridge imaging phenotype with tumor biology, supporting standardized and biologically grounded risk models. These approaches remain exploratory and should not yet be interpreted as validated clinical decision tools [53].

## 7. Artificial Intelligence and Advanced Computational Approaches

Interobserver variability, lesion conspicuity differences, and discordance between mpMRI and PSMA PET interpretations remain important limitations [100]. Emerging AI-based approaches may help mitigate these challenges by integrating multiparametric imaging features into reproducible predictive models. Radiomics-based analyses combining ADC metrics and PSMA uptake parameters have demonstrated potential for improved lesion classification and aggressiveness prediction [101]. Deep learning frameworks trained on multimodal imaging datasets may further enhance automated lesion detection, segmentation, and staging accuracy [102].

Nevertheless, AI-driven models remain largely investigational as challenges include small training datasets, lack of harmonized imaging acquisition protocols, variability in feature extraction pipelines, and limited external validation [103]. Before clinical implementation, standardized reporting frameworks and prospective multicenter validation are required.

## 8. Conclusions

Integrating mpMRI with PSMA PET can improve prostate cancer imaging and treatment. PSMA PET focuses on nodal, distal, and early recurrence. In contrast, mpMRI can precisely characterize local tumors, evaluate extracapsular expansion, and guide targeted biopsies. This integrated technique could improve surgical planning, radiation targeting, and systemic therapy by accurately identifying tumor load. Quantitative biomarkers from both modalities enable precision medicine and reduce over- or undertreatment by assessing risk individually. Standardizing imaging procedures, validating findings through prospective studies, and integrating imaging with molecular and genetic markers will improve individualized therapeutic regimens. The combination of mpMRI with PSMA PET revolutionizes precision imaging, improving diagnostic accuracy, therapeutic choices, and patient management.

## Figures and Tables

**Table 1:** Quantitative Imaging Biomarkers and Clinical Correlates. Overview of established and emerging quantitative imaging biomarkers from mpMRI and PSMA PET and their reported correlations with tumor aggressiveness, burden, and risk stratification.

Modality	Biomarker	Clinical Correlation
mpMRI	ADCmin	Gleason grade, aggressiveness
PSMA PET	SUVmax	Tumor burden, progression
Hybrid	ADC + SUV	Improved risk prediction

**Table 2:** Comparative Strengths and Limitations of mpMRI vs. PSMA PET. Comparison of the principal diagnostic capabilities, quantitative biomarkers, and limitations of mpMRI and PSMA PET, highlighting their complementary roles in local tumor characterization and whole-body staging.

Feature	mpMRI	PSMA PET
Primary tumor detection	Excellent (T2/DWI/ADC)	Moderate
Local staging (ECE, SVI)	Excellent	Limited
Nodal metastases	Limited (size-based)	Excellent
Distant metastases	Poor	Excellent
Quantitative biomarkers	ADC	SUV, TBR
Limitations	TZ lesions, inter-reader variability	False positives, PSMA-negative tumors

**Table 3:** Clinical Impact of Integrated mpMRI + PSMA PET by Disease State. Summary of key clinical scenarios in which combined mpMRI and PSMA PET imaging improves risk stratification, disease localization, and treatment decision-making, supported by representative studies.

Clinical Scenario	Added Value of Integration	Representative Evidence
**Biopsy-naïve patients**	Improved risk stratification	[7]
**Intermediate-risk PCa**	Occult nodal detection	[67]
**Biochemical recurrence**	Localization at PSA < 0.5	[69]
**Radiotherapy planning**	Field modification	[70]

## Data Availability

No new data were created or analyzed in this study. Data sharing is not applicable to this article.

## Supplementary Materials

The following supporting information can be downloaded at: https://www.mdpi.com/article/10.3390/medicina62030610/s1, Supplementary Table S1. Database-Specific Search Strategies.
