# Supplementary material for: Integrating Multiparametric MRI and PSMA PET Imaging in Prostate Cancer: Toward a Unified Diagnostic and Risk-Stratification Paradigm
Source: Medicina (Kaunas). 2026 Mar 23;62(3):610. doi: 10.3390/medicina62030610 (PMC13027863; doi:10.3390/medicina62030610)
Supplement: Supplementary file 1 [file medicina-62-00610-s001.zip › medicina-4151202-supplementary.pdf]

Supplementary Table S1.

Database	Search Strategy (keywords and Boolean operators)
**PubMed / MEDLINE**	("prostate cancer"[Mesh] OR "prostate neoplasms"[Mesh] OR "prostate cancer"[tiab] OR "prostate carcinoma"[tiab]) AND ("multiparametric MRI"[tiab] OR "mpMRI"[tiab] OR "magnetic resonance imaging"[Mesh]) AND ("PSMA PET"[tiab] OR "prostate-specific membrane antigen"[tiab] OR "PSMA positron emission tomography"[tiab]) AND (diagnosis[tiab] OR staging[tiab] OR risk stratification[tiab] OR recurrence[tiab] OR treatment planning[tiab]) Filters: Publication date from January 2015 to January 2026; English language.
**Embase**	('prostate cancer'/exp OR 'prostate neoplasm*':ab,ti OR 'prostate carcinoma':ab,ti) AND ('multiparametric mri':ab,ti OR 'magnetic resonance imaging'/exp) AND ('psma pet':ab,ti OR 'prostate specific membrane antigen':ab,ti OR 'positron emission tomography'/exp) AND (diagnosis:ab,ti OR staging:ab,ti OR 'risk stratification':ab,ti OR recurrence:ab,ti OR 'treatment planning':ab,ti) Limits: 2015–2026; English language.
**Web of Science**	TS=("prostate cancer" OR "prostate carcinoma" OR "prostate neoplasm*") AND TS=("multiparametric MRI" OR "mpMRI" OR "magnetic resonance imaging") AND TS=("PSMA PET" OR "prostate-specific membrane antigen" OR "PSMA positron emission tomography") AND TS=(diagnosis OR staging OR "risk stratification" OR recurrence OR "treatment planning") Timespan: January 2015–January 2026; English language.

Supplementary Table S1. Database-Specific Search Strategies
